# Clinical and Genetic Characterization of 269 Patients With Suspected Inherited Platelet Disorders: The Padua Monocentric Experience

**DOI:** 10.1111/ijlh.70081

**Published:** 2026-02-23

**Authors:** Silvia Ferrari, Daniela Regazzo, Antonella Bertomoro, Anna Cerbo, Alessandro Galbiati, Elisabetta Cosi, Paolo Simioni

**Affiliations:** ^1^ Department of Medicine DIMED Padova University Hospital Padova Italy

**Keywords:** inherited platelet disorder, molecular analysis, platelets, thrombocytopenia

## Abstract

**Background:**

Inherited platelet disorders (IPDs) are rare hematologic conditions encompassing a heterogeneous spectrum of quantitative and qualitative platelet defects, frequently associated with variable clinical phenotypes and comorbidities. Accurate diagnosis necessitates comprehensive genetic characterization, detailed clinical and bleeding history, and systematic evaluation of platelet count, size, and function, which are essential to distinguish IPDs from immune thrombocytopenia (ITP).

**Aim:**

The aim of this study was to clinically and genetically characterize a total of 269 patients from 140 families with platelet disorders, evaluated at the First Chair, Department of Medicine, University of Padua.

**Methods:**

Patients with suspected platelet disorders underwent comprehensive laboratory evaluation, and Sanger sequencing was performed to identify causative variants in approximately 30% of cases.

**Results:**

We identified 80 patients from 44 families who were found to carry pathogenic or likely pathogenic variants, with the most frequent ones being linked to Bernard‐Soulier syndrome (BSS), characterized by thrombocytopenia and bleeding tendency. Additionally, 23 subjects had *MYH9* gene variants and one patient was affected by Glanzmann thrombasthenia. We also discovered seven pathogenic variants not previously described in the literature.

**Conclusion:**

Due to the limitations of the Sanger method, the molecular defect could only be defined in approximately 30% of cases. In the remaining 70% of patients, the genetic cause remained unidentified, highlighting the need for further evaluation using an extended NGS panel targeting genes associated with inherited thrombocytopenias.

## 1 Introduction

Inherited platelet disorders (IPDs) are a group of rare genetic conditions characterized by abnormalities in platelet number and/or function, resulting in bleeding tendencies of variable severity. These may present as qualitative defects affecting platelet function with a normal platelet count, quantitative defects leading to thrombocytopenia, or a combination of both.

More specifically, inherited thrombocytopenias (ITs) represent a subset of IPDs in which decreased platelet count is the hallmark, sometimes accompanied by functional platelet defects. Understanding the genetic and clinical heterogeneity of these disorders is essential for accurate diagnosis and management.

*IPDs* are considered a rare disease, but their frequency is likely underestimated due to diagnostic challenges that require expertise often limited to specialized centers [1].

The majority of patients exhibit a mild bleeding tendency, which may not be significantly different from that of healthy individuals. This explains why platelet defects are frequently discovered incidentally in adulthood, with many patients initially misdiagnosed as having immune thrombocytopenia (ITP) [2].

In the past two decades, advances in genetic characterization techniques, such as NGS, have led to the identification of nearly 50 different IPD forms, involving numerous genes [3, 4].

Interestingly, since 2010, 14 novel IPD‐associated disorders have been described, demonstrating that IT is far less rare than previously thought, affecting at least 2.7 per 100.000 individuals [2].

Recent advancements in the molecular diagnosis of IPDs have enabled their classification into syndromic and non‐syndromic forms. Moreover, certain IPDs are associated with an increased risk of hematologic malignancies [5].

However, it is beyond the scope of this work to describe all known forms of IPDs. Therefore, we will limit our discussion to those that are both the most frequent and relevant to the patients analyzed in the present cohort. In this section, we report the most common forms identified through our clinical experience.

Among IPDs‐related forms, the most common and well‐known is the classical, biallelic form of Bernard‐Soulier Syndrome (BSS), a disorder affecting 1:1.000.000 individuals and characterized by recurrent, life‐threatening hemorrhages [6, 7]. Beyond BSS, other inherited platelet disorders have been increasingly recognized, including MYH9 gene‐associated inherited thrombocytopenia (IT). The prevalence of *MYH9* gene‐associated IT is estimated at around 3:1.000.000, based on data from an Italian registry [8, 9, 10].

Glanzmann thrombasthenia (GT) is also a rare IPD, with an estimated worldwide prevalence of approximately one in a million. However, due to its autosomal recessive inheritance, prevalence is higher in populations with high consanguinity [11, 12]. GT generally does not involve thrombocytopenia; however, a GT‐like phenotype with reduced platelet count has been described in association with heterozygous variations in *ITGB3* and *ITGA2B* [13].

Germline variants in *ANKRD26*, *RUNX1*, and *ETV6* have been identified as causative factors for IPDs. These forms predispose affected individuals to an increased risk of hematological malignancies [14, 15, 16, 17, 18]. The identification of *ANKRD26* in 2013 as the gene responsible for a mild form of autosomal‐dominant thrombocytopenia, with reduced platelet α‐granules, normal platelet aggregation, and normal platelet size, led to an international collaborative study revealing that this disorder exposes individuals to the risk of myelodysplastic syndromes and acute myeloid leukemia [16].

Pathogenic heterozygous variants in the *RUNX1* gene represent an established cause of mild to moderate thrombocytopenia, functional and ultrastructural platelet defects, and predisposition to myelodysplastic syndrome and acute myeloid leukemia [15, 17]. In addition, in recent years, several heterozygous germline mutations in *ETV6* have been associated with familial thrombocytopenia and predisposition to hematologic malignancies. Mutations or abnormalities in the *ETV6* gene have been linked to several pathological conditions, including certain types of leukemia, such as acute myeloid leukemia and acute lymphoblastic leukemia [14, 18].

In 2014–2015, *SLFN14*‐related thrombocytopenia (SLFN14‐RT) was described as a non‐syndromic thrombocytopenia associated with an excessive bleeding phenotype and defective platelet ATP secretion [19].

During the same period, mutations in the *ACTN1* gene causing ACTN1‐related thrombocytopenia (ACTN1‐RT) were reported. This autosomal‐dominant form is caused by mutations in the *ACTN1* gene which encodes one of the two non‐muscle isoforms of α‐actinin [20].

In the era of extensive genetic sequencing and the identification of newly recognized IPDs‐associated genes, identifying patients with IPDs predisposed to additional disorders is still essential in order to personalize follow‐up, predict the risk of recurrence, and provide appropriate treatments if new illnesses develop. In this context, alongside genetic characterization, an accurate study of the platelet size, platelet function testing, and the quantitative analysis of receptors by flow cytometry can provide further insight into genetic findings.

In many laboratories, an initial approach using the Sanger method to characterize the genetic etiology of IPDs remains the first step for identifying the gene defect in the more frequent hereditary IPDs forms, especially in cases with defined clinical suspicion. It also remains the molecular approach to confirm variants found through NGS analysis. Furthermore, NGS in a research setting remains an essential tool for the identification of new variants or new genes involved in the platelet defects, helping to obtain a clear diagnosis through the analysis of multigene panels [21, 22, 23].

The application of NGS, including whole exome sequencing, has greatly accelerated the discovery of causative genes and expanded the list of variants in more common disorders. However, not all the existing forms have yet been characterized, and less than approximately 40% of patients remain without a definite diagnosis [24, 25].

The development of a diagnostic algorithm that incorporates simple diagnostic tests, which can be used even in less specialized centers, may facilitate a more accurate diagnosis of platelet defects [26, 27].

In this contest, clinical assessment is a critical first step in the diagnostic algorithm to differentiate syndromic from non‐syndromic platelet disorders. It involves a comprehensive review of a patient's medical information, including a bleeding assessment and other histories, such as cardiovascular diseases, hematopoiesis status, history of malignancy, neuromuscular diseases, vision issues, and renal disease.

Focusing on bleeding assessment, reliably establishing the bleeding phenotypes of patients and their family members, the International Society of Thrombosis and Hemostasis (ISTH) bleeding assessment tools (BAT) are recommended to calculate a bleeding score for accurate evaluation and to standardize the assessment of bleeding severity [28].

In addition to the ISTH‐BAT, the WHO (World Health Organization) bleeding scale might also be used. This tool allows for the classification of the clinical severity of bleeding episodes on a scale from 0 to 4 and is particularly useful in the assessment of acute bleeding or in therapeutic contexts [29].

Moreover, the standard laboratory tests include complete blood count (CBC), platelet morphology, peripheral blood smear PFA‐100, 200 (Platelet Function Analyzer) and platelet function tests including platelet aggregation (Born method), and platelet immunophenotype (by flow cytometry) to define the platelet phenotypic defects. The results of these tests can help confirm the clinical suspicions and also provide phenotypic data before proceeding with genetic tests.

Keeping these assumptions in mind, the aim of this study was to collect and analyze clinical and genetic data from 269 patients and their relatives, evaluated over approximately 20 years at the First Chair of Internal Medicine, University Hospital of Padua. Our goal was to investigate the genetic basis of platelet defects, characterize phenotypic patterns, and correlate clinical and laboratory findings with genetic results.

## 2 Material and Methods

### 2.1 Design and Methods

Patients were studied in accordance with the Declaration of Helsinki, and its ethical standards and all subjects gave their written informed consent.

A total of 269 patients belonging to 140 families with suspected (or with family histories) of IPDs was collected and investigated between 2000 and 2024 at the First Chair of Internal Medicine at University‐Hospital of Padua.

For each patient, comprehensive data were collected, including detailed medical history, bleeding phenotype, genotype, family history of thrombocytopenia, sex, age at onset, platelet count, mean platelet volume (MPV), obstetric history, platelet aggregation assays, flow cytometry results, and genetic analyses.

### 2.2 Bleeding Tendency

The bleeding tendency was assessed using both the World Health Organization (WHO) bleeding scale and the ISTH Bleeding Assessment Tool (ISTH‐BAT). The WHO scale classifies bleeding severity as follows: grade 0, no bleeding; grade 1, petechiae; grade 2, mild blood loss; grade 3, gross blood loss; and grade 4, debilitating blood loss. The ISTH‐BAT, a validated quantitative questionnaire, was used to systematically evaluate the patient's bleeding history across multiple domains, providing a cumulative bleeding score indicative of an underlying platelet disorder [29].

For the sake of consistency and continuity in data analysis, we opted to continue using the WHO bleeding scale, as it was employed at the onset of the study. Nevertheless, we fully acknowledge the robustness and clinical relevance of the ISTH Bleeding Assessment Tool (ISTH‐BAT).

### 2.3 Platelet Count

EDTA tube (ethylenediaminetetraacetic acid) were used for platelet counting on the Sysmex XP‐300 automated hematology analyzer (Sysmex Corporation, Kobe, Japan). Instrument parameters were set according to the manufacturer's instructions.

### 2.4 Platelet Size

The platelet diameter was measured by optical microscopy on May Grünwald Giemsa stained peripheral blood films, and observed with an optical microscope (Leica DMR) with 100X immersion objective.

The percentage of large platelets on the total number of platelets, evaluated during the observation with the optical microscope after staining, corresponds to the percentage of platelets whose diameter is equal to or greater than half of a red blood cell (normal values: 1%–6%).

### 2.5 Evaluation of the Presence of Antiplatelets Antibody

To rule out immune thrombocytopenia (ITP), platelet‐bound anti‐platelet antibodies were detected by flow cytometric analysis of platelet‐rich plasma (PRP) [30].

To obtain PRP, sodium citrate anticoagulated blood was centrifuged at 110 rcf for 10 min without brake, and the obtained separated plasma was collected.

When the patients was negative for ITP and based on platelet morphology, IT was suspected, and immunophenotype characterization of platelet glycoproteins was performed by flow cytometry, at the same time with Born aggregation test followed by genetic testing. Immunophenotype characterization, born aggregation, and genetic testing are described above.

### 2.6 Platelet Immunophenotype Characterization

Single 22.5 μL aliquots of PRP were incubated with fluorescein‐labeled monoclonal antibody against CD41 (GPIIb/IIIa‐complex), CD42a (GPIX), CD42b (GPIbα) (Coulter, Immunotech, Marseille, France), CD49b (GPIa), activated GPIIb/IIIa using PAC1 after stimulation of platelet with ADP (10 μM), CD62P selectin (Beckman Coulter, Germany), and analyzed using FC 500 Flow Cytometer (Beckman Coulter, Germany), and cytometer settings were reported by Bausset et al. [31].

### 2.7 Platelet Aggregation

Platelet aggregometry (Born method) was performed on PRP using a Chrono‐log aggregometer Mascia Brunelli, Milano. The following agonists were applied: collagen 2 μg/mL (N.V. 72%–98%), adenosine diphosphate (ADP) 2 μM (N.V. 60%–100%) and 10 μM (N.V. 60%–100%), ristocetin 1.5 mg/mL (N.V. 77%–100%), and adrenaline 10 μM (N.V. 62%–92%). All agonists were purchased from Mascia Brunelli.

### 2.8 Isolation and Sequencing of Genomic DNA

Genomic DNA was isolated from sodium citrate blood samples using the QIAampDNA Mini Kit (Qiagen, Hilden, Germany). The genes *GP1BA* (exon 1, NM_000173.7), *GP1BB* (exons 1 and 2, NM_000407.5), *GP9* (exons 1, 2, 3, NM_000174.5), *MYH9* (exons 1, 10, 16, 24, 25, 26, 30, 38, 40, NM_002473.6) and *ANKRD26* (5′UTR region, NM_014915.3), *RUNX1* (exons from 1 to 6, NM_001754.5), *ETV6* (exons from 1 to 8, NM_001987.5), *SLFN14* (exons from 1 to 6, NM_001129820.2), *ACTN1* (exons from 1 to 22, NM_001130004.2) were amplified by PCR. The primer sequences and PCR conditions are available upon request. PCR fragments were prepared for sequencing reaction by using Exostar (Illustra) and sequenced using the BigDye Terminator cycle Sequencing V3.1 (Applied Biosystems) following manufacturer's instruction and analyzed on an ABI3130 genetic analyzer (Applied Biosystems, Foster City, Ca, USA).

In our analysis, we did not detect any variants in the *SLFN14, ACTN1*, and *ETV6* genes.

All the variant were classified accordingly to ACMG criteria. The pathogenicity of the detected variants was assessed according to ACMG/AMP guidelines Richards et al. [32]. Multiple lines of evidence were integrated, including population frequency data (e.g., PM2, BA1), functional studies (PS3/BS3), segregation analysis in available family members (PP1/BS4), in silico predictions (PP3/BP4), and previously reported clinical data (PS1/PP5). Variants were classified as pathogenic, likely pathogenic, or of uncertain significance based on the combination of these criteria.

### 2.9 Statistical Analyses

Categorical variables are reported as frequency with percentage, and continuous variables as medians with interquartile ranges (IQRs), unless otherwise specified.

## 3 Results

### 3.1 Clinical Picture

A total of 269 patients, belonging to 140 families, were referred to our Unit, First Chair of Internal Medicine at University‐Hospital of Padua, primarily from Northeastern Italy.

Of these, 141 patients were female (53%) and 128 patients were male (47%). The age range of the study group was from 3 to 80 years (median = 28).

For female subjects, when available, we collected information regarding their pregnancies, partum management, hemorrhage, therapy types, and the presence of thrombocytopenia in the newborns.

Seventeen women with IPDs had a total of 35 children. Among these, 12 were diagnosed with *BSS*, 9 with *MYH9‐RD*, and 5 with *ANKRD26‐RD*. Thirteen of the women underwent caesarean sections, one had an abortion, and three required platelet transfusions during delivery. According to detailed anamnesis, 9 children exhibited the same platelet defects as their mothers. One child died from a brain hemorrhage, but no child showed visible bleeding at birth.

In addition, we reported the presence of 3 patients with hearing loss, one with cataracts and renal failure, and one patient with a transplanted kidney, features associated with the clinical picture of *MYH9‐RD*. All subjects were negative for anti‐platelet antibodies.

### 3.2 Genetic Analysis

Genetic analysis was performed for all 268 patients with suspected platelet disorder.

**FIGURE 1:**
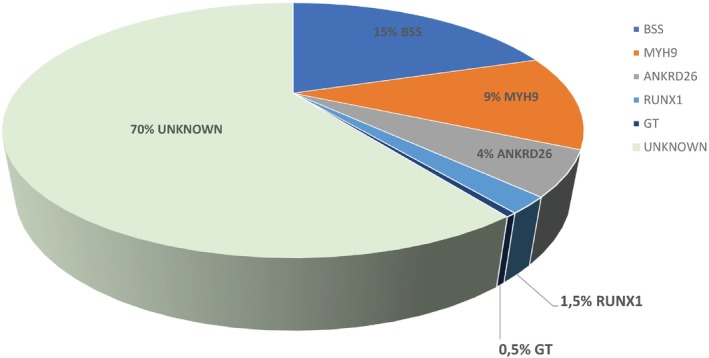
Relative frequencies of various forms of inherited thrombocytopenias in a total of 269 patients. ANKRD26, ankyrin repeat domain containing 26; BSS, Bernard Soulier syndrome; GT, glanzmann thrombasthenia; MYH9, myosin heavy chain 9; RUNX1, RUNX family transcription factor 1.

In 80 patients from 44 families, a genetic mutation was identified. Among these, 41 patients had pathogenic variants in genes related to the monoallelic form of the BSS, 23 had *MYH9* gene defects, 11 had *ANKRD26* genetic variants, and 4 were affected by *RUNX1* genetic alterations. All genetic variations are reported in (Table 1 and Figure 1).

**TABLE 1:** Genetic variant found in each family.

Family	*N* of tested members (carriers/healthy member)	*Gene*	Variant names in mature protein	Status	Variant class
Family 1	2 (1/1)	*GP1BA*	NM_000173: c.515C>T; p.Ala172Val	HET	P
Family 2	8 (4/4)	*GP1BA*	NM_000173: c.515C>T; p.Ala172Val	HET	P
Family 3	3 (3/0)	*GP1BA*	NM_000173: c.515C>T; p.Ala172Val	HET	P
Family 4	3 (2/1)	*GP1BA*	NM_000173: c.814G>T; p.Val272Phe	HET	P
Family 5	2 (2/0)	*GP1BA*	NM_000173: c.171C>A; p.Asn57Lys	HET	P
Family 6	26 (9/17)	*GP1BA*	NM_000173: c.169A>C; p.Asn57His	HET	P
Family 7	3 (2/1)	*GP1BA*	NM_000173: c.217C>T; p.Leu57Phe	HET	P
Family 8	17 (4/13)	*GP1BA*	NM_000173: c.169 A>C; p.Asn57His	HET	P
Family 9	1 (1/0)	*GP1BA*	NM_000173: c.376A>G; p.Asn126Asp	HOM	P
Family 10	1 (1/0)	*GP1BA*	NM_000173: c.515C>T; p.Ala172Val	HET	P
Family 11	1 (1/0)	*GP1BA*	NM_000173: c.217C>T; p.Leu73Phe	HET	P
Family 12	1 (1/0)	*GP1BA*	NM_000173: c.1523delAT; p.Asp508Valfs*19	HET	LP
Family 13	3 (3/0)	*GP9*	NM_000174: c.70T>C; p.Cys24Arg	HET	P
Family 14	2 (2/0)	*GP1BB*	NM_000407: c.179T>C; p.Leu60Pro	HET	LP
Family 15	2 (2/0)	*GP1BB*	NM_000407: c.455C>T; p.Ala152Val	HET	P
Family 16	1 (1/0)	*GP1BB*	NM_000407: c.179T>C; p.Leu60Pro	HET	LP
Family 17	3 (2/1)	*GP1BB + GP9*	NM_000407: c.389C>T; p.Pro130Leu: NM_000174: c.442_443insG; p.Val148Glyfs Ter67	HET + HOM	LP
Family 18	2 (2/0)	*MYH9*	NM_002473: c.5521G>A; p.Glu1841Lys	HET	P
Family 19	3 (1/2)	*MYH9*	NM_002473: c.287C>T; p.Ser96Leu	HET	P
Family 20	1 (1/0)	*MYH9*	NM_002473: c.2104C>T; p.Arg702Cys	HET	P
Family 21	8 (6/2)	*MYH9*	NM_002473: c.5797C>T; p.Arg1933Ter	HET	P
Family 22	7 (2/5)	*MYH9*	NM_002473: c.5521G>A; p.Glu1841Lys	HET	P
Family 23	4 (4/0)	*MYH9*	NM_002473: c.5797C>T; p.Arg1933Ter	HET	P
Family 24	2 (2/0)	*MYH9*	NM_002473: c.3485G>C; p.Arg1162Thr	HET	P
Family 25	3 (1/2)	*MYH9*	NM_002473: c.5521G>A; p.Glu1841Lys	HET	P
Family 26	1 (1/0)	*MYH9*	NM_002473: c.4270G>C; p.Asp1424His	HET	P
Family 27	1 (1/0)	*MYH9*	NM_002473: c.5797C>T; p.Arg1933Ter	HET	P
Family 28	4 (1/3)	*MYH9*	NM_002473: c.5797C>T; p.Arg1933Ter	HET	P
Family 29	1 (1/0)	*MYH9*	NM_002473: c.3485G>C; p.Arg1162Thr	HET	P
Family 30	2 (1/1)	*ANKRD26*	NM_014915: c.‐128G>C, 5′UTR	HET	P
Family 31	1 (1/0)	*ANKRD26*	NM_014915: c.‐140C>G, 5′UTR	HET	LP
Family 32	1 (1/0)	*ANKRD26*	NM_014915: c.‐140C>G, 5′UTR	HET	LP
Family 33	1 (1/0)	*ANKRD26*	NM_014915: c.‐140C>G, 5′UTR	HET	LP
Family 34	1 (1/0)	*ANKRD26*	NM_014915: c.‐140C>G, 5′UTR	HET	LP
Family 35	1 (1/0)	*ANKRD26*	NM_014915: c.‐140C>G, 5′UTR	HET	LP
Family 36	1 (1/0)	*ANKRD26*	NM_014915: c.‐140C>G, 5′UTR	HET	LP
Family 37	1 (1/0)	*ANKRD26*	NM_014915: c.‐140C>G, 5′UTR	HET	LP
Family 38	1 (1/0)	*ANKRD26*	NM_014915: c.‐140C>G; c.‐134G>A, 5′UTR	Compound HET	LP
Family 39	1 (1/0)	*ANKRD26*	NM_014915: c.‐134G>A, 5′UTR	HET	LP
Family 40	1 (1/0)	*GT*	n.a.	n.a.	n.a.
Family 41	1 (1/0)	*RUNX1*	NM_001754: c.308C>G; p.Pro103Arg	HET	LP
Family 42	1 (1/0)	*RUNX1*	NM_001754: c.316T>G; p.Trp106Gly	HET	LP
Family 43	1 (1/0)	*RUNX1*	NM_001754: c.1379delA p.(Ser460ThrfsTer134)	HET	LP
Family 44	1 (1/0)	*RUNX1*	NM_001754: c.1658_1660insTG	HET	LP

Abbreviations: Het, heterozygous; Hom, homozygous; LP, like pathogenetic; P, pathogenetic.

Subjects in whom no genetic variants were identified also exhibited mild thrombocytopenia (100 × 10^9^/L–150 × 10^9^/L), which was the reason for their referral to our center; no signs of bleeding were observed in any patient. Additionally, about 20% of subjects without genetic variants presented with large platelets.

No patients with MYH9‐RD, ANKRD26, RUNX1‐RD, or patients without genetic alterations showed any abnormalities in platelet aggregation or immunophenotype compared to the normal pattern.

Glanzmann thrombasthenia (GT) was diagnosed without genetic analysis, based on the clinical phenotype. Platelet aggregation was severely reduced in response to ADP, collagen, thrombin, and epinephrine, while the response to ristocetin was normal. Immunophenotyping showed a reduced expression of CD41 (GPIIb), and the platelet count was normal.

### 3.3 Bleeding Tendency

Sixty‐eight patients (85%) had no bleeding history (Grade 0). Grade 1 was found in 7 patients (9%) and Grade 2 and 3 in both 2 patients respectively (2.5%), and one patient was classified as Grade 4 (1%). The most common symptoms were epistaxis, gum bleeding, easy bruising, and menorrhagia. Two patients with Grade 3 showed epistaxis and gum bleeding that required medical intervention. The Grade 4 patient showed severe and multiple bleeding episodes, including post‐partum hemorrhage, and required a platelet transfusion.

### 3.4 Platelet Count

Thrombocytopenia values in *BSS* was typically mild (median 65 × 10^9^/L, IQR 50–86). Patients with *MYH9‐RD* had a lower mean platelet count (median 44 × 10^9^/L, IQR 31.5–49.5). A median platelet count of 69 × 10^9^/L (IQR 41–112) was observed in patients with *ANKRD26* gene defect. The GT patient had a platelet count of 200 × 10^9^/L, and the patient with RUNX1‐RD had a median platelet count of 82.5 × 10^9^/L (IQR 66–99.75).

### 3.5 Platelet Size

Mean platelet volume (MPV) was found to be increased in patients with BSS, showing values of 16% (normal range 8%–12%). In patients with *MYH9*‐RD, MPV was even more elevated, attesting to a value of 26%. In contrast, patients with IT caused by other genes, or with no identified genetic defect, had MPV values within the normal range.

### 3.6 Immunophenotype Characterization

After the analysis of the platelet immunophenotype of the 17 subjects with BSS, CD42b expression was reduced in 10 subjects with *GP1BA* genetic variants and in 2 with *GP1BB* alteration, while 1 subject with a pathogenic variant in *GP9* had reduced expression of both CD42b and CD42a.

The subject suspected having GT showed no CD41a (GPIIb/IIIa) and PAC1 (activated GPIIb/IIIa) surface expression.

### 3.7 Platelet Aggregation

In all 41 BSS patients (proband and affected relatives) the platelet agglutination induced by ristocetin (1.5 mg/mL) was absent. Aggregation with collagen (2 μg/mL), adrenaline (10 μM), and ADP at a low dose (2 μM) and high dose (10 μM) was normal.

The single GT subject had a normal platelet count and morphology but showed strikingly defective aggregation to multiple agonists (ADP, collagen). Ristocetin agglutination was normal or moderately decreased in the second wave. No alterations in platelet aggregation were observed in patients with other genetic defects or in patients with no identified genetic defect.

## 4 Discussion

This study aimed to collect, examine, and analyze the clinical and diagnostic data of 269 patients who, over a span of 20 years, sought evaluation at the First Chair of Internal Medicine at the University of Padua Medical School for suspected platelet disorders. By reconstructing the clinical history, assessing bleeding tendencies, and characterizing platelet function in detail for each patient, we contributed to a more precise understanding of the pathophysiology of these rare disorders, where novel genetic variants are frequently identified.

Consistent with the literature [33], bleeding manifestations in IPDs can range from mild to severe, often presenting with mucocutaneous bleeding symptoms such as epistaxis, bruising, and menorrhagia. Among them, bleeding in inherited thrombocytopenia is generally mild or absent, with less frequent or milder hemorrhagic manifestations.

In our cohort, 85% of cases, calculated among the 80 patients carrying pathogenic or likely pathogenic variants, presented with Grade 0, and pregnancy outcomes in women with IPDs were comparable to those of the general population. Notably, more than 90% of these women did not require transfusions of blood products.

During the diagnostic process, we found that platelet count assessment and peripheral blood smear analysis were essential as rapid and simple screening tools to guide further laboratory investigations. In the majority of cases where large or giant platelets were observed, this finding was indicative of macrothrombocytopenia associated with platelet glycoprotein defects or *MYH9‐RD*.

Platelet morphology findings obtained through optical microscopy were further supported by flow cytometry‐based platelet immunophenotyping. Flow cytometry proved particularly useful in identifying platelet glycoprotein expression defects in Bernard‐Soulier Syndrome (BSS), as subjects with BSS exhibited reduced CD42b (*GP1BA*) or CD42a (*GP9*) expression. Platelet aggregation studies revealed a reduced or absent response to ristocetin, confirming the characteristic defect of BSS, in agreement with previous literature [34]. This analysis was performed in all 23 BSS patients.

Molecular analysis revealed that the most frequent IPD in our cohort was monoallelic BSS, with the p.Asn57His variant in *GP1BA* identified in 13 individuals, making it the most frequent variant, with no significant sex‐based differences. This was followed by the p.Ala172Val variant in *GP1BA*, found in 9 subjects (Table 1). Both variants have been previously described and are predicted to be likely pathogenic [35].

A novel *GP1BB* variant, p.Leu60Pro, was identified, which has not been reported in public databases but is likely pathogenic [36]. Additionally, in another BSS patient, we identified a potential compound pathogenic effect involving a novel homozygous frameshift variant p.Val148GlyfsTer, in the transmembrane region of *GP9*, in combination with a p.Pro130Leu missense variant in *GP1BB* [37]. These variants are reported here for the first time by our group.

Among patients with *MYH9*‐related thrombocytopenia and giant platelets, the most frequently observed variant was p.Arg1933Ter, detected in 12 individuals. This variant introduces a premature stop codon and has been previously established as pathogenic in the literature [38]. No novel genetic variants were identified in the *MYH9* gene within our cohort.

Genetic analysis was particularly instrumental in identifying patients with IPDs presenting with normal platelet size but an elevated risk of hematologic malignancies, such as those with *RUNX1* and *ANKRD26*‐related thrombocytopenia. Pathogenic variants in these genes were detected in 8% of our subjects [16]. In prior studies [39, 40], we identified the c.‐140 C>G variant in the 5′UTR of *ANKRD26*, and we further contributed to understanding the role of 5′UTR variants by identifying c.‐128 G>A and c.‐134 G>A. As also reported in the literature, we observed that the vast majority of families with individuals carrying variants in the *ANKRD26* gene had at least one member affected by a solid tumor or hematological malignancy [16].

In addition to *ANKRD26*‐related thrombocytopenia, we identified novel likely pathogenic variants in the *RUNX1* gene, which is well‐known for its role in hematologic malignancies [15]. For one of these variants, we also conducted an expression study [17].

Genetic testing in this study proved invaluable for confirming IPD diagnoses in cases where phenotypic laboratory results were inconclusive or unavailable. It also facilitated carrier detection in family members, enabling early identification and management of affected individuals. Genetic testing was particularly advantageous when platelet aggregation and immunophenotyping results are nonspecific, making definitive diagnosis challenging.

Despite the advancements in genetic testing, significant gaps remain in our understanding of these rare platelet disorders. Approximately 70% of cases in our cohort remained genetically unresolved when analyzed by Sanger sequencing alone (Figure 1). Recognizing the limitations of Sanger sequencing, the next step involves applying an in‐house designed multigene NGS panel, comprising 85 genes implicated in platelet dysfunction that may help in increasing diagnostic yield in our cohort. IPD‐focused NGS panels enable simultaneous sequencing of multiple genes associated with specific phenotypes, thereby improving diagnostic yield. The ultimate goal of further studies is to elucidate the molecular profile of patients who exhibit complex clinical phenotypes consistent with IPDs, but for whom no genetic defect was identified in the genes analyzed to date. Future research efforts will focus on these unresolved cases to enhance our understanding of IPD pathogenesis and improve diagnostic precision.

## Author Contributions

**Silvia Ferrari:** performed the research, designed the research study, analysed the data, and wrote the paper. **Daniela Regazzo:** analysed the data and wrote the paper. **Antonella Bertomoro:** analysed the data. **Anna Cerbo:** analysed the data. **Alessandro Galbiati:** analysed the data. **Elisabetta Cosi:** analysed the data. **Paolo Simioni:** analysed the data and contributed essential reagents or tools.

## Funding

The authors have nothing to report.

## Ethics Statement

AOP 3786; CET‐ACEV: 6310/AO/25.

## Consent

Patients were studied in accordance with the Declaration of Helsinki, and its ethical standards.

## Conflicts of Interest

The authors declare no conflicts of interest.

## Data Availability

The data that support the findings of this study are available from the corresponding author upon reasonable request.
